# Durability of Hydrophobic/Icephobic Coatings in Protection of Lightweight Concrete with Waste Aggregate

**DOI:** 10.3390/ma14010101

**Published:** 2020-12-29

**Authors:** Danuta Barnat-Hunek, Jacek Góra, Marcin K. Widomski

**Affiliations:** 1 Faculty of Civil Engineering and Architecture, Lublin University of Technology, Nadbystrzycka 40, 20-618 Lublin, Poland; d.barnat-hunek@pollub.pl (D.B.-H.); j.gora@pollub.pl (J.G.); 2 Faculty of Environmental Engineering, Lublin University of Technology, Nadbystrzycka 40 B, 20-618 Lublin, Poland

**Keywords:** lightweight concrete, waste polystyrene, hydrophobization, icephobic coating, silane, absorptivity, contact angle, freezing and thawing cycles, durability

## Abstract

The aim of the research presented in this paper is to evaluate the feasibility of using hydrophobic agents based on organosilicon compounds for surface protection of lightweight concrete modified with waste polystyrene. The experimental part pertains to the physical and mechanical properties of polystyrene-modified lightweight concrete. The concrete samples were prepared with the following ingredients: CEM I 42.5 R cement, recycled polystyrene (0–2 mm), quartz sand (0–2 mm), coarse river aggregate (2–16 mm), and water. Silane and tetramethoxysilane were employed for surface hydrophobization. Concrete with 20% polystyrene exhibits high porosity (25.22%), which is related to an increase in absorptivity (14.75%) compared to the reference concrete. The hydrophobized concrete is characterized by the lowest surface free energy (SFE) value, which is 7 or 11 times lower than the value of reference concrete, depending on the agents. The test on the contact angle (CA) was performed before and after the frost-resistance test (F–T test). Lower SFE translates into lower adhesive properties, higher resistance of the material to the infiltration of water and corrosive compounds, e.g., salts, and higher resistance to freezing and thawing cycles. Silane and tetramethoxysilane coating raised frost resistance by 54–58% compared to the reference samples. This agent reduced absorptivity by 30%. Recycled polystyrene can be successfully used to produce lightweight concrete (LC) with high durability provided by hydrophobic/icephobic coatings.

## 1. Introduction

Construction and demolition waste is rapidly increasing in developed countries. The low biodegradability of polymers, the base of numerous building materials, has triggered numerous economic and ecological problems. One of the EU’s priorities is reaching 50% recycling by 2025. Significant financial fees are threatened in the case of failure [1]. Recently, in relation to the increasing problem of waste use, engineers are looking for possible reuse methods. One is the reintroduction of waste as components of the energy-efficient construction materials [2]. With the rapid development of energy-efficient buildings, lightweight concrete (LC) has become a modern, promising construction material [3,4,5,6].

In comparison to the standard concrete, LC has very good characteristics, such as lower density and thermal conductivity and higher energy absorption due to the partial or total replacement of standard aggregates with lightweight coarse aggregates [7]. The lightweight artificial aggregates, obtained from industrial waste, having undergone heat treating or fragmentation, may be used for LC production. Artificial lightweight aggregates obtained from building material recycling and possible for use in LC may be expanded clay aggregates [8], pumice aggregate [9], perlite [10,11,12], Styrofoam/polystyrene waste [13,14,15], granulated cinder [16], polyurethane waste [17], and waste expanded cork [18]. Research by Degirmenci et al. [19] showed that compressive strength of mortar containing pumice, after cyclic freezing and thawing, was greater than mortar containing Portland cement, thus application of pumice caused greater frost resistance.

Recycled polystyrene may be also used as aggregate in LC production. Expanded polystyrene (EPS), thermoplastic resin, is a type of artificial aggregate of density 10–30 kg/m^3^ [20], which may be obtained through the recycling of polystyrene plates during the demolition or renovation of buildings. Polystyrene on an industrial scale can be obtained by the method of suspension polymerization. Polystyrene granules increase volume by 65 times; Styrofoam consists of 98% air, and only 2% polystyrene [21]. Thus, it is ultralight, and its mechanical characteristics depend on density. Styrofoam is safe for human health: non-dusting, non-allergic, non-radioactive, chemically neutral, and resistant to mold and fungi.

Compared to a very low density of polystyrene, the concrete mixture is prone to component segregation. There have been several studies that have focused on the elimination of this problem, including the addition of silica powder to improve adhesion between EPS aggregates and cement paste. Roy et al. [22] used superplasticizer to avoid the segregation of EPS particles. Chen and Liu [23] applied latex of styrene-butadiene rubber (SBR) to improve the durability of concrete. 

Recycled Styrofoam may partially solve this problem, due to its unique pitted, strongly rough, uneven structure, obtained during mechanical fragmentation, which may significantly increase the adhesion of concrete to Styrofoam. Additionally, Styrofoam shows good sorption characteristics and may accumulate enough ice and salt in its structure. However, porous Styrofoam in open structures may cause increased water absorptivity by concrete [21]. Moreover, the preparation of concrete in winter conditions may cause fractures and a reduction of concrete strength, due to the maturation in low temperatures [22]. Thus, the protection of concrete structures, endangered by frost, is crucial for reducing the deterioration speed and improvement in durability [24].

Surface hydrophobization is one of the protection methods for concrete against frost corrosion and salts dissolved in water [25,26,27]. Organosilicon compounds are commonly used in concrete hydrophobization. The basis of organosilicon polymers is siloxane Si–O–Si and silica-carbon Si–C–Si bonds [28]. Very good efficiency of mortars and LC was reported by Ramachandran et al. [29], Szafraniec et al. [30], Suchorab et al. [8], Liu et al. [31], and Barnat-Hunek et al. [32], or Sadowski and Stefaniuk [33]. 

It was showed that the hydrophobization of LC composites containing recycled polyurethane foam increased frost resistance by over 80%, compared to the reference samples [34]. However, surface hydrophobization is not always efficient in the improvement of concrete characteristics, especially when the concrete contains industrial waste [24,30,35]. Studies performed by Liu et al. [31] proved that surface hydrophobization of concrete by silane reduces damage caused by cycles of freezing and thawing, but is unable to prevent damage inside insufficiently aerated concrete. Therefore, silane forms an impervious cover on the surface of the concrete, limiting water infiltration and constricting water vapor diffusion outside, causing structural damage [31]. Similar conclusions were reported by different authors, especially for cases in which particles of polysiloxane resign were too large to penetrate the tiny porous structure of concrete [32,35]. Łukowski [36] and other researchers [28,34,35] confirmed that the geometry and composition of the polymer particle, not the chemical nature of polymer compound, had significant relevance during the formation of the polymer coating. As a result of water penetration thorough the pervious coating and frost activity, stratification at the joint of the concrete base and the polymer coating is possible. If hydrophobic coating is insufficient for protecting concrete against penetration by water, due to its underdevelopment or mechanical damage, especially cracking, then hydrophobization may cause an increased destruction process compared to unimpregnated material. 

Hydrophobization of concrete results in a shift of the ice crystallization zone into the depth of the material. Thus, the adhesion of coating to the concrete base ensures the efficiency of hydrophobization [28,35]. Regarding the low-molecular structure of silanes, such formulations, e.g., low-molecular alkyl-alkoxy-siloxane, showed very good penetration capabilities and reacted chemically inside the concrete in the presence of atmospheric moisture into a hydrophobic active substance resistant to external factors—polysiloxane [21,28,35]. In the case of wide-porous materials, such as ceramsite concrete, the highest efficiency of hydrophobization was obtained by the application of macromolecular methyl-silicone resins [8,35].

The negative effects of hydrophobization on the physico-mechanical characteristics of LC causes the need for further studies concerning this problem. In this paper, the problem of building material recycling, including Styrofoam, is considered. As a result, the application of Styrofoam aggregates in LC is proposed. 

To the best of our knowledge, there are no examples available in the literature of a hydrophobizing agent such as silane and a new-generation agent such as tetramethoxysilane being applied in LC with polystyrene aggregates from recycling. There are papers presenting the use of polystyrene in concrete, but not recycled polystyrene, which has a different, rough, and jagged structure. So far, we have been unable to find any paper reporting surface hydrophobization of such concretes. Therefore, this study focuses on the evaluation of the concrete with recycled polystyrene surface modification.

This paper presents the physico-mechanical characteristics of lightweight concrete with the addition of recycled Styrofoam, and comparative analysis of the effect of hydrophobic/icephobic coatings based on organosilicon compounds (silane and tetramethoxysilane) on the properties of heat LC with polystyrene aggregates from recycling. The influence of hydrophobization agents on CA and frost resistance were tested particularly carefully. The values of the contact angle of concrete by the singular test liquid were determined before and after frost-resistance tests. Then, surface free energy (SFE), characterizing wetting and water adhesion to hydrophobic coatings under standard conditions and after cyclic exposure to low temperatures, was determined by the Neumann method. The structure of LC and the adhesion of cement paste to the polystyrene aggregates were presented with the application of the scanning electron microscope (SEM) technique. As a result, the hydrophobization efficiency of LC containing polystyrene is determined. 

## 2. Materials and Method 

### 2.1. Materials

The presented studies covered three concrete specimens: reference concrete without recycled polystyrene (LC0), concrete with 10% (LC10), and 20% (LC20) addition by volume of recycled polystyrene (EPS). The addition, which was used to partially replace the volume of sand according to its particle size distribution, was assessed as the waste aggregate of particle size 0–2 mm. All studied concrete samples underwent surface hydrophobization with two different agents: silane (A1) and tetramethoxysilane (A2). Both applied hydrophobizing agents were produced by CFI World S.A. 62-023 Robakowo, Poland, based on ingredients from Evonik Industries, Business Line Silanes, Rodenbacher Chaussee 4 63457 Hanau-Wolfgang, Germany. Trade names of used preparations are as follows: A1—Protectosil^®^ WS 405; A2—Protectosil^®^ WS 808. The manufacturer does not provide data on A1—water emulsion based on organofunctional silanes. Silane is an inorganic compound with chemical formula SiH_4_, making it a Group 14 hydride. Surface hydrophobization was carried out by applying the agent twice with a brush using the “wet-on-wet” method.

The properties of applied hydrophobizing agents are presented in Table 1. 

Natural washed quartz sand, 0–2 mm, of particle sizes compliant to applied waste aggregate (EPS), and natural gravel, 2–16 mm, was used in the concrete sample preparation. The main component of the applied waste aggregate (EPS) is polystyrene. The chemical composition of the quartz sand is SiO_2_ 95.3%, Al_2_O_3_ 1.9%, Fe_2_O_3_ 0.7%, and CaO 0.35% [18]. 

Natural gravel quartz from sandstone and minerals in gravel grains was derived from plutonic rocks—orthoclase and albite—minerals in the grains were derived from sedimentary rocks—calcite, dolomite, and illite. The bulk density of the used sand and gravel was the same, i.e., 2.65 kg/dm^3^, while the bulk density of the EPS addition was definitely lower, i.e., 0.018 kg/dm^3^. Portland cement CEM I 42.5 R from a cement plant in Chełm, Poland (CEMEX Polska sp. z o.o.) was used in all the studied concrete samples. The cement producer also delivered detailed characteristics of the supplied cement. Chemical composition of the used cement clinker is presented in Table 2, while physical the properties of the cement are shown in Table 3.

To compare influence of waste aggregate content on the properties of concrete, the same volume of fine aggregates in 1 m^3^ of concrete was assumed across all tested mixtures. The particle size distribution of the fine and coarse aggregates is shown in Table 4.

All concrete mixtures used in this study had the same water/cement ratio, i.e., w/c = 0.40. The comparable consistency of all concrete samples was obtained due to the application of superplasticizer based on polycarboxylic ether. First, the dry components were mixed slowly in a mixer for 2 min. Then, slowly and gradually, water was added to the mixture to obtain accurate connection and to avoid segregation of the mixture components. The mixing continued for 5 min. The concrete mixture was compacted in molds on the vibration table with oscillations frequency 50 Hz to obtain full compaction, without excessive segregation and excretion of cement milk. The samples after demolding were cured in water of temperature 20 ± 5 °C. The performed concrete slump test for all concrete mixtures indicated 12 ± 2 cm, which reflects consistency class S3 [36]. The compositions of the tested concretes containing recycled polystyrene are presented in Table 5. 

### 2.2. Method

The volumetric density of the tested concretes was determined according to EN 12390-7:2019 standard [37] on 6 cubic samples with edges 150 mm long. Specific density was tested by pycnometer method after grinding the concretes in a ball mill into particles of dimensions lower than 0.08 mm. The air included between the grinded particles of the tested materials was removed by placing the pycnometers into a vacuum chamber, and pressure reduction to the value of 2.33 kPa in a vacuum chamber by Memmert GmbH + Co.KG, Schwabach, Germany. The total porosity of the studied concretes was calculated using values of volumetric and specific density.

The compressive strength of tested specimens was determined on 6 cubic samples with edges 150 mm long according to EN 12390-3 [38]. The flexural strength during bending was measured on beams of dimensions 100 × 100 × 500 mm according to EN 12390-5 standard [39]. Tests of compressive and flexural strength were performed after 28 days of sample maturation in water of temperature 20 ± 2 °C. Strength measurements of all tested concrete samples were performed in a computer-controlled testing machine (allowing for constant stress increase) by Walter + Bai AG, Löhningen, Switzerland.

The mass absorptivity of water was determined on cubic samples of edge length 150 mm, according to PN-B-06250 [40]. The concrete samples for absorptivity tests were placed on a grid 10 mm above a reservoir bottom and covered with water to ¼ of their height for 24 h. The water table was raised after each 24 h to ½ and ¾ of sample height. After the next 24 h, the water level in the testing reservoir was elevated up to 10 mm over the samples. Then, after an assumed duration, the samples were removed from the reservoir and, after wiping all their surfaces, they were weighted. Mass absorptivity was determined after 1, 7, and 14 days of curing of the samples. After the end of saturation, the tested samples were dried to a dry mass at temperature of 105 ± 5 °C and weighted. 

To determine the frost resistance of the studied concretes, F–T tests were performed, according to PN-B-06250 [40], based on 50 freeze and thaw cycles. The mass and compressive strength of 150 mm cubic samples were measured. For each F–T test, 12 samples were prepared. Six were placed in the chamber for frost-resistance tests and underwent cycling freezing and thawing. The remaining 6 samples (reference samples) were kept for the test duration in water of temperature 20 ± 2 °C. Freezing was performed for 4 h at a temperature of −20 ± 2 °C, then the samples were thawed in water for 4 h until reaching a temperature of +20 ± 2 °C. The mass reduction of freezing samples and decrease in compressive strength were assessed compared to values observed for reference samples. Frost-resistance tests for all studied concrete samples were performed in the computer-controlled chamber, produced by Elektromechanika Chłodnicza G. Skibiński, Poland, for cyclic material freezing and thawing, allowing a constant temperature and determined time of freezing and thawing. 

The water vapor permeability of the tested concrete samples was determined by the cup method according to EN ISO 7783:2012 [41]. Cylindrical samples of diameter 100 mm and 20 mm height were prepared. Two layers of hydrophobizing agent was placed on one surface of the samples. The hydrophobic coating was under atmospheric pressure. The tested sample in the cup was placed inside the climatic chamber KK115 (POL-EKO-APARATURA sp.j., Wodzisław Śląski, Poland) to sustain the required relative humidity. Measurements were taken under conditions of constant temperature 22 ± 2 °C and relative humidity of 93 ± 2% and 50 ± 2% for both sides of the samples, respectively. The samples were placed in cups containing a saturated water solution of NaCl on one side. The humidity inside the cup was assured by a saturated solution of (NH_4_)_2_HPO_4_. The samples were regularly weighted due to a loss of moisture until reaching a state of equilibrium. The experiment took 8–10 days, regarding water vapor permeability of the tested material.

The measurements of the contact angle, describing characteristics of liquid drop, were performed by goniometer (OCA 15EC Data Physics Instruments GmbH, Filderstadt, Germany) and camera for the precise photography of drops on the surface of concrete samples. All the studied concretes were included in the measurements, before and after frost-resistance tests. The measurements were conducted in distilled water, because of its high polarity, at a temperature of 22 ± 2 °C. Six drops of distilled water of volume approximately 2 mm^3^ were applied on the surface of each sample by pipette. SFE was determined by Neumann method [8,18,35] using Formula (1) [42].

(1) $$cos\theta_{w}=\left[e^{-0.000125{\left(\gamma_{s}-\gamma_{w}\right)}^{2}}2\sqrt{\frac{\gamma_{s}}{\gamma_{w}}}-1\right]$$

where: *θ_w_*—water contact angle (°), $\gamma_{s}$—total SFE (mJ/mm^2^), and $\gamma_{w}$—SFE of liquid (distilled water) = 72.8 (mJ/mm^2^).

Research concerning the resistance of concrete against salt crystallization was conducted according the standard PN-EN:12370 [43]. 14% solution of decahydrate sodium sulfate was used in each test. Each concrete sample, dried to the dry mass, was placed in a lockable container and covered with sodium solution with elevation 8 ± 2 mm. Next, the container was sealed shut. The samples were left at a temperature of 20 ± 2 °C for 2 h, then the samples were dried at a temperature of 105 ± 5 °C for 16 h. To achieve relatively high humidity, an oven was heated to a constant temperature through time duration not shorter than 10 h. Before the repeated insertion of samples into the sodium solution, the samples were left for 2 h at room temperature to decrease temperature. The test covered 6 reference samples and 6 samples of each concrete hydrophobized by two applied agents (A1 and A2). The measurements covered 15 cycles, then samples were cleaned and dehydrated to the dry mass. 

The microstructure of the tested concretes was observed with a scanning electron microscope (SEM Quanta FEG 250 microscope by Field Electron and Ion (FEI), Hillsboro, OR, USA). The working distance was set as 10–13 mm. Next, to achieve conductivity on the surface of the samples, they were covered with a 45 nm layer of carbon. Such treatment excludes the possibility of micro-defects due to concrete surface and cracking. Low vacuum and low beam energy were applied during observations to avoid the possibility of other defects of the sample surface. 

## 3. Results and Discussion

### 3.1. Basic Properties of Lightweight Concrete

The results of basic physical and strength tests of the considered LCs are presented in Table 6. 

According to the EPS content, the tested concrete samples showed different values of volumetric density, starting from 2080 kg/m^3^ for LC20 with 20% EPS content to 2250 kg/m^3^ for LC0. The volumetric porosity is 2 and 3.9 times greater after partial replacement of sand by EPS in the amount of 10 and 20% by volume, respectively. The above is reflected in the results of compressive and flexural tensile strength measurements for tested samples. 

The compressive strength *f*_c,cube#150_ after EPS addition decreased by 10.2% and 18.4% for LC10 and LC20, respectively. However, the flexural tensile strength *f*_ct,flex_ decreased by 13.5% for LC10 and by 32.3% for LC20. Cadere et al. [44] noticed that addition of polystyrene instead of sand caused a reduction to the compressive strength of between 47.7% and 75.4%, compared to EPS content in the studied concrete. On the other hand, split tensile strength of concrete with polystyrene decreased by 14.5–44.2% [44]. 

Research by Ranjbar and Mousavi [45] showed that the compressive strength of self-compacted LC containing expanded polystyrene decreased from 60 MPa for standard concrete without EPS of w/b = 0.38 to 34 and 25 MPa for samples of concrete containing 10% and 22.5% EPS, respectively. Despite the observed decrease in the durability of concrete containing EPS, it should be noted that Sussman and Baumann [46] noticed that in the case of the even value of density, the concrete containing EPS presented 70–270% higher compressive strength than concrete containing vermiculite and perlite aggregates. 

**Figure 1:**
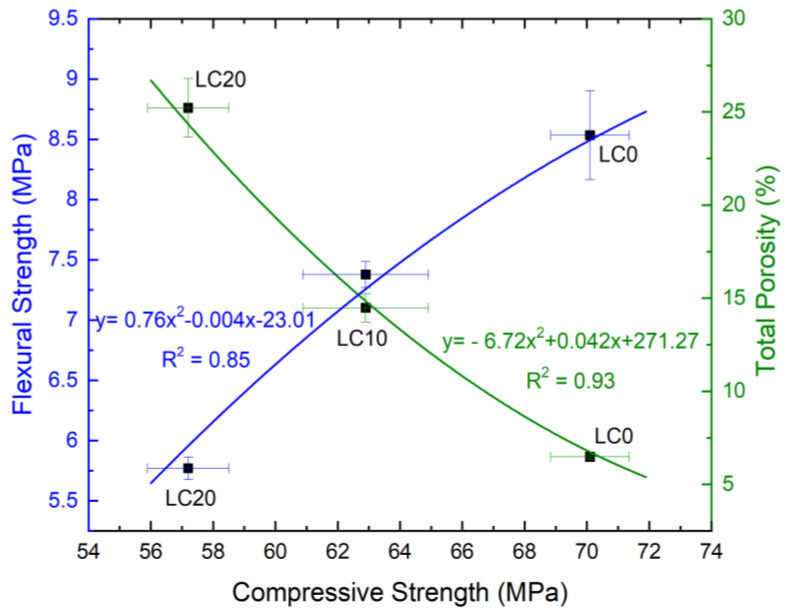
Correlation between compressive strength, flexural tensile strength, and total porosity.

The correlation between the compressive strength *f*_c,cube#150_ and flexural tensile strength *f*_ct,flex_ as well as the correlation between compressive strength and total porosity are shown in Figure 1.

As can be observed in Figure 1, the flexural strength value is strongly linked to the compressive strength of the concretes. The flexural strength increases along the increase of compressive strength, according to equation y = 0.76x^2^ − 0.004x − 24.01. Values of both analyzed strength characteristics decrease with the increase in EPS content in concrete. On the other hand, the opposite relationship was observed between compressive strength of concrete and its total porosity: the higher porosity, the lower compressive strength. In this case, a very strong correlation (r = 0.96, R^2^ = 0.93) was observed. 

### 3.2. Properties of Lightweight Concrete after Surface Hydrophobization 

**Figure 2:**
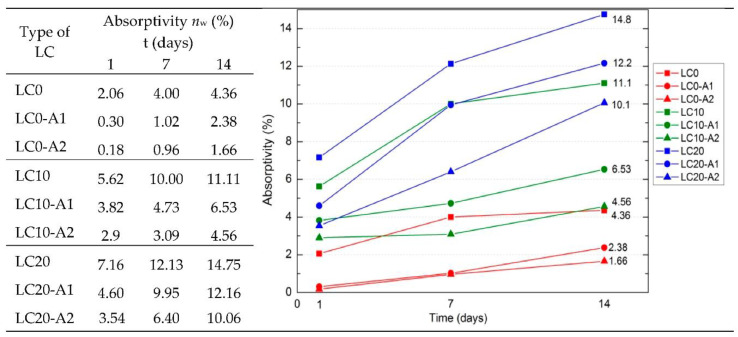
Water absorptivity *n*_w_ by weight of LC in time (%).

Figure 2 presents the determined LC average water absorptivity *n*_w_ by weight values after 1, 7, and 14 days of testing. As can be noted, the applied surface hydrophobization clearly reduced concrete absorptivity. The best results of absorptivity reduction were obtained for concretes LC0-A1 and LC0-A2. After the first day of testing, absorptivity decreased by 11 times and after 14 days by 2.6 times, compared to the standard LC0 samples. The highest value of determined absorptivity was noted for LC20 concrete, containing a greater amount of EPS, also after hydrophobization. The absorptivity of LC20-A1 is 5 times, and of LC20-A2 is 6 times higher than for the hydrophobized standard concrete samples LC0-A1 and LC0-A2. Higher hydrophobization efficiency for all tested series of concrete was obtained due application of A2, a macromolecular tetramethoxysilane compound. This formulation probably created an impervious coating on the surface of the concrete. The efficiency of the hydrophobization process is mainly dependent on the porosity of the concrete. Along with the increase in porosity, the absorptivity of LC increases, and hydrophobization becomes more effective because the applied agent may penetrate the structure of the concrete. 

As presented by Błaszczyński et al. [47], efficient and durable protection requires the agent to provide high permeability to the concrete construction and a high content of active substance; in this case, the agent may penetrate deeper layers of the protected material. It was also noted that hydrophobization was weaker (less durable) in the case of impervious concrete, because the hydrophobization formulation was unable to penetrate the composite structure, in contrast to materials of higher porosity. Numerous studies have reported the positive influence of hydrophobization on the absorptivity and permeability of cement materials [8,21,24,26,29,30,31,32,33,34]. 

**Figure 3:**
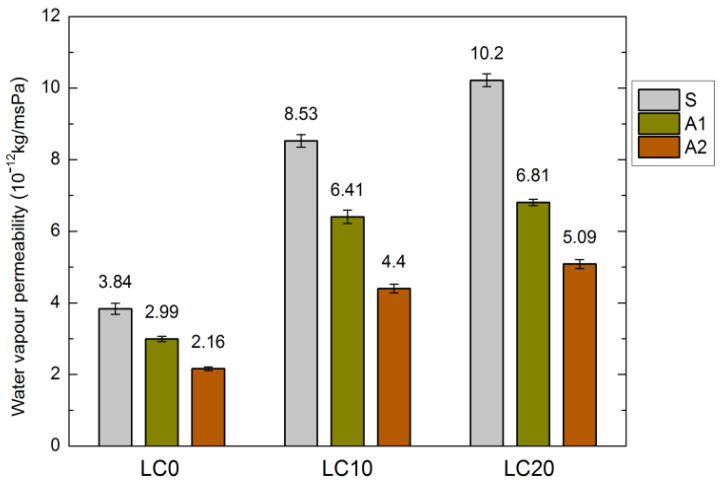
Water vapor permeability of LC before and after hydrophobization (10^−12^ kg/msPa).

Water absorption is strongly related to the water vapor permeability of LC, which is greatest for the LC20 samples. The results of measurements of the LC water vapor permeability, before and after hydrophobization, are presented in Figure 3. The hydrophobic coating should reduce the water absorptivity of the concrete, but it should also allow penetration of water vapor through the concrete pores. As mentioned in several papers [21,28,35], hydrophobization may constrict evaporation. 

The structure of the tested concrete samples was slightly sealed during the applied hydrophobization process. The diffusion of water vapor was most strongly distorted by tetramethoxysilane (A2). In the cases of LC10-A2 and LC20-A2, the water vapor permeability is twice as low as that for the standard concrete samples. The above is related to the size of the resin particles, which sealed the fine surface pores of concrete. Sabatini et al. [48] reported that water vapor permeability decreased for all tested samples of the hydrophobized cement composites. Similar results were obtained by Barnat-Hunek et al. [49], who determined a 3–4-fold decrease in water vapor permeability of hydrophobized mortars. 

The strongly sealed coating, significantly reducing the water vapor permeability of concrete, may trigger damage in the material structure, mainly due to salt and ice crystallization [21,32,35]. The equilibrium between blocking and increasing the unsaturated moisture flow is highly related to the compatibility of the pore structure and the size of polymer particles in hydrophobization formulation [50]. 

The other characteristic related to water absorption and water vapor permeability is the water CA. The values of CA measured using a goniometer before and after the F–T cycles are shown in Table 7.

The results of the tests presented in Table 7 indicate that the determined values of water CA depend on the type of concrete and, in particular, EPS content. Along with the increase in EPS content in concrete and the increase in absorptivity, the CA decreases, and is 22.7% lower for LC10 ad 93% for LC20, respectively, compared to values determined for LC0. All the tested LCs are hydrophilic; their measured CD was lower than 90°. The hydrophilicity of LCs increases along with the increase in the content of the absorbent EPS. The greater porosity and absorbability of the concrete causes a decrease in the adhesion forces between the concrete surface and water. This is a natural phenomenon. Hydrophobization performed with A1 and A2 agents caused a significant increase in the hydrophobicity of the studied concretes. The application of the A2 agent on LC0 and LC10 LCs allowed for superhydrophobic surfaces, with CA greater than 125° [35]. During previous studies [35], the greatest CA values were noted for reference mortar without waste aggregate before (39.7°) and after hydrophobization (113.4°) with new-generation nanopolymers—trietoxiisobutylsilan. One of the principal parameters with an influence on hydrophobization efficiency is the adhesion of siloxane films to the substrate. All the analyzed concrete samples were successfully protected from the effect of frost. However, during the F–T cycles, delamination may occur at the contact spot between the material and film, as a results of the infiltration of water through a polysiloxane gel film. Such a situation occurred in the analyzed concretes. There are known papers, including the authors’ own works, that indicate that hydrophobic coatings that covered the concrete too tightly resulted in excessive ice crystallization pressure, which severely damaged the aggregates and the contact spot between the aggregates and the cement paste [1,35,51]. Then, the surface becomes more absorbent and the CA decreases, which was also observed during our research [35,51].

The clear decrease in determined values of water CA was visible after F–T tests indicated reduced efficiency of hydrophobization under the influence of frost. The decrease in CA was in the range 23–23% for LC0-A2 and LC10-A2, respectively. In the case of the reference concrete L0, the applied coating kept its hydrophobic properties, and the determined CAs values were equal to 103° and 110° for A1 and A2, respectively. All the tested concrete samples containing EPS lost their hydrophobic properties. Thus, in practice, highly absorptive concrete surfaces should be cyclically hydrophobized each year or every few years as needed, just as in the case of the analyzed LC10 and LC20. 

Najduchowska and Pichniarczyk [52] proved in their research that standard cement mortar was characterized by a high water contact angle of approximately 37°. After hydrophobization with organosilicon compounds containing methyl groups, the determined CA reached the level of 101°, which was related to the type of applied agent. The tests of the water CA and frost resistance of hydrophobized mortars with polystyrene aggregate, reported by [31], showed a two-fold decrease in the measured values of reference mortars CA after 25 cycles of F–T. The hydrophobized samples studied in [31] showed significantly higher frost resistance. The observed CA was reduced by 3.5% with application of methyl-silicone resin and 9% with alkyl-alkoxy-silane (AAS). 

**Figure 4:**
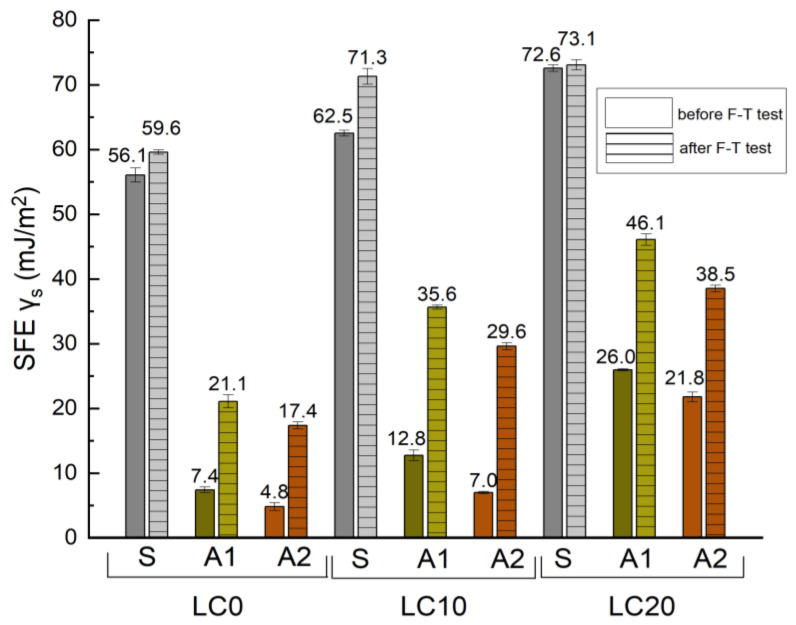
SFE values of LC before and after F–T cycles.

The SFE is a key factor in the assessment of the physico-chemical properties of a solid’s surface. The surface may present a dispersive (dispersive component) or polar (polar component) character. According to characteristics of the impregnating agents, it is possible to increase or decrease the SFE, and therefore the surface tension of the materials causing their non-wettability, which is related to resistance to chemical corrosion and frost. The greatest decrease in SFE may be caused by coatings with the highest degree of hydrophobization of the surface [21,28,35]. The values of the determined SFE of the referenced and hydrophobized concretes, based on measured CA and calculated by the Neuman method, are presented in Figure 4. 

Analyzing the results presented in Figure 4, it may be assumed that the determined SFE values are dependent on the type of hydrophobizing agents. The lowest value of SFE, thus the weakest adhesive properties, was determined for LC hydrophobized by tetramethoxysilane A2 (4.59, 6.88, and 21.85 mJ/m^2^ for LC0, LC10, and LC15, respectively, before frost-resistance tests, and 17.11, 29.87, and 38.56 mJ/m^2^ after frost-resistance tests). In the case of LC0-A2 the determined value of SFE is 92% lower than for LC0-S. After frost-resistance tests, all values of SFE increased, which was related to the decrease in measured CA (see Table 7). In the case of hydrophobic agent application, values of SFE increased by approximately three times, which indicates a decrease in the hydrophobic properties of the studied coatings. 

The lowest values of SFE tested in [32] characterized the reference mortar without lightweight waste (SFE = 59.6 mJ/m^2^), which, after hydrophobization, dropped to 15.1 mJ/m^2^. The highest decrease in SFE, 70%, was observed for mortar containing 10% waste polyurethane foam, PVC, and wood chips after hydrophobization by a nanopolymer-based agent. Król et al. [53] used silane coatings to modify the wettability of materials. In this case, SFE value was lower than 3 mJ/m^2^, while in the studies performed by Barnat-Hunek et al. [51] a value of SFE ranging from 10–37 mJ/m^2^ were obtained for polysiloxane coatings. An increase in SFE values after frost-resistance tests was observed, which indicates a decrease in hydrophobization efficiency and damage to the hydrophobic coatings. 

The determined values of SFE increased by 73%, 77% 64%, and 87% for LC0-A2, LC10-A2, LC10-A1, and reference concrete LC0, respectively. Crystallization of ice under the hydrophobic coating caused damage in its continuity, which leads to greater wettability (affected CA and SFE). The similar observations were reported by [28] after tests of SFE for hydrophobized bricks undergoing several F–T cycles. The introduction of organosilicon compounds into the near-surface zone of the concrete caused, regarding the chemical composition of the applied agents, a reduction of SFE and surface tension of the concrete, influencing a reduction of entry to the corrosive substances into the structure of the concrete, thus increasing its durability. 

**Figure 5:**
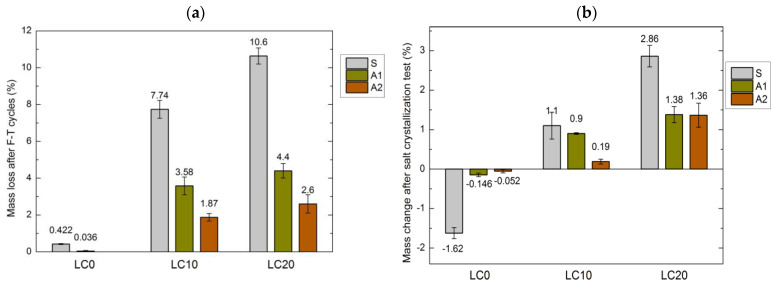
Mass change of LC: (**a**) after F–T cycles, (**b**) after salt crystallization test.

To assess the durability of hydrophobized concrete samples containing EPS, the research concerning the negative influence on tested concrete of frost and salts dissolved in water was performed. The determined decrease in mass of concrete samples after 50 F–T cycles is presented in Figure 5a, while the determined mass loss after 15 cycles of drying and wetting in decahydrate solution of Na_2_SO_4_ is presented in Figure 5b.

The determined results of the frost resistance of the tested concrete samples allowed the determination of the relationship between mass loss and the other characteristics of concrete. The susceptibility of LC to damages caused by F–T cycles is related to concrete composition, its porosity, and, primarily, the type of applied hydrophobizing agent. The EPS content, decreasing sealing, and frost resistance seems to have a significant influence. The freezing water increasing its volume tends to fill the empty pores of concrete. 

According to Fagerlund [54], this process causes an increase in water pressure inside the porous structure of the concrete. As stated by Pigeon and Pleau [55], if the force of ice expansion exceeds the flexural strength of concrete, microcracks appear. Then, a greater volume of water penetrates the concrete and the damage caused by processes of F–T cycles is more serious.

As predicted, significant damage in the EPS concrete structure was observed. The frost resistance of LC10 and LC20 after 50 cycles of F–T decreased by 18 and 25 times, respectively. Similar observations were reported by Barnat-Hunek et al. [11] after tests of self-compacting LC with perlite aggregate. Richardson et al. [56], Shang et al. [57], and Yildirim et al. [58] studied the influence of recycled aggregates on the frost resistance of concrete. The obtained results showed that along with the increase in the number of F–T cycles, the strength of the concrete decreased as a result of damage to the interphase transition zone (ITZ) between aggregates and cement paste. 

A significant improvement was obtained after application of A1 and A2 coatings on all the tested concrete types. The studies showed that surface hydrophobization significantly slows the creation of damage, microcracks etc. and protects the concrete against destruction during F–T cycles (see Figure 5a). The tetramethoxysilane agent A2 reduced the strength of both concrete samples by over 75%, and silane by 54% and 58% in the cases of LC10 and LC20, respectively. There was no clear decrease in the strength of the reference concrete LC0. 

The best frost resistance was determined by [32] for a nanopolymer agent that improved this characteristic by 71–89%, compared to the recycled aggregate content. However, the application of polysiloxy resin reduced mass loss by 50% for mortar containing a 10% amendment of polyurethane foam and wood shavings. Studies concerning frost resistance of hydrophobized cement mortar with polystyrene [21] showed that methyl-silicone resin caused an increase in frost resistance by 62% and low-molecular-weight alkyl-alkoxy-siloxane by 78% in relation to reference mortar samples. The fine particle compound was able to penetrate deeper the structure of material deeper, which caused better efficiency of hydrophobization than macromolecular resin.

The other factor influencing the strength and durability of concrete is resistance to aggressive ion penetration [35,59]. Sodium sulfate is considered to be the most damaging salt that has two hydrated phases in ambient conditions, i.e., a stable decahydrate called mirabilite, and a metastable heptahydrate (Na_2_SO_4_ × 10H_2_O and Na_2_SO_4_ × 7H_2_O). Damage is usually caused by direct mirabilite crystallization or by the dissolving of heptahydrate [60].

Scherer [61] and Mancigotti and Hamilton [60] proved that the pressure of salt crystallization is lower in greater pores. Thus, the destructive influence of salt crystallization is stronger in small pores [59]. The salt particle increases its volume during moisture absorption from 5 to 10 times, depending on its type, triggering an internal stress of range 100–200 MPa. This may result not only in the separation of the hydrophobic coating but also may lead to the destruction of the concrete material below the surface layer [35].

**Figure 6:**
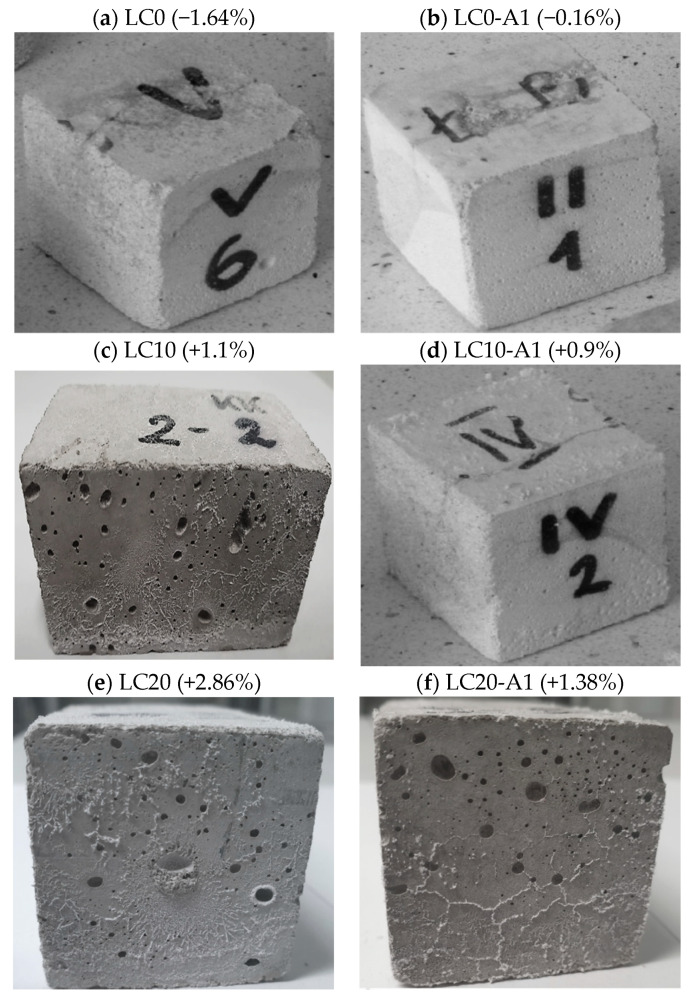
Samples after salt crystallization test. (**a**) LC0 (−1.64%); (**b**) LC0-A1 (−0.16%); (**c**) LC10 (+1.1%); (**d**) LC10-A1 (+0.9%); (**e**) LC20 (+2.86%); (**f**) LC20-A1 (+1.38%).

Figure 6 presents the condition of the cubic samples of the LC following an increase/decrease in sample mass after the salt crystallization test.

Hydrophobization of the LC surface was unable to protect the samples from salt outgrowth, which can be observed from visible white coating on the sample surfaces (Figure 6d,f). The reference concrete LC0, due to a limited volume of pores, was slightly damaged (1.62% of mass reduction) (see Figure 6b). After hydrophobization by silane (A1), the mass loss was 11 times lower, and after that by tetramethoxysilane (A2), 31 times lower. The increase of EPS by 20% caused an increase in sample mass by 2.86% and 1.38% after hydrophobization. These concrete samples contain porous, rough additions in which the unwanted process of salt crystallization occurred. There was no damage to concrete with 10% and 20% of volumetric EPS content. 

Studies performed by Szafraniec et al. [30] showed that hydrophobization of mortar containing perlite with water emulsion based on silanes caused a decrease in sample mass loss by up to 18 times. Additionally, an increase in the concentration of hydrophobic agents caused a reduction in mass loss after the test of resistance against salt crystallization. Research by Suleiman et al. [62] proved the efficiency of silanes in the surface protection of concrete against harmful sulfate corrosion. None of the tested concrete samples showed mass changes or surface damages. Silane infiltrates the near-surface pores of concrete, limiting capillary rise and preventing salt crystallization inside the pores. Studies performed by Barnat-Hunek et al. [49] showed that alkyl-alkoxy-siloxane effectively prevents surface damage to mortar containing expanded cork. The dissolved salt crystallized inside the samples without causing any damage. A very good resistance to salt crystallization was observed for plaster containing zeolites, ceramsite, and slag after hydrophobization by silanes [16]. 

**Figure 7:**
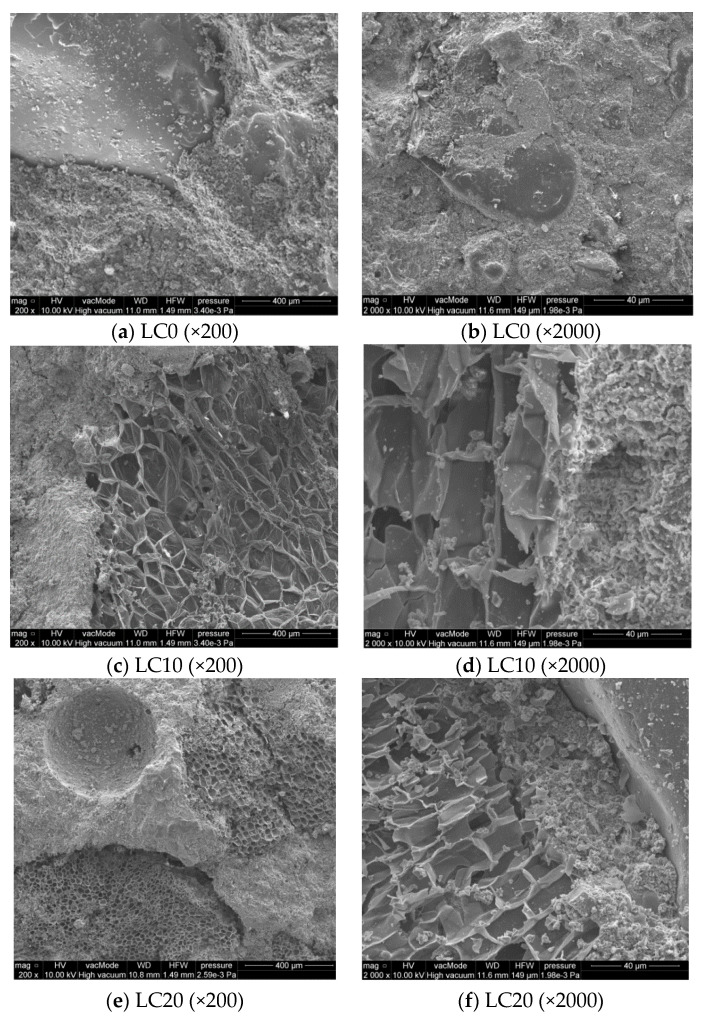
Internal microstructure of LC without/with EPS. (**a**) LC0 (×200); (**b**) LC0 (×2000); (**c**) LC10 (×200); (**d**) LC10 (×2000); (**e**) LC20 (×200); (**f**) LC20 (×2000).

Morphologic (SEM) observations of selected LC samples were performed to show their structure, ITZ between EPS and cement paste, and the possible cracks. Figure 7 presents the microstructure and adhesion of the cement paste to EPS and sand.

Figure 7a,b shows the compact structure of low-porosity LC0 concrete. The remaining SEM pictures (Figure 7c–f) show LCs with EPS addition in which the structure is clearly porous, with sharp and torn edges as a result of mechanical processing of recycled polystyrene. The observation of ITZ between paste cement and EPS showed the filled area in close-up (Figure 7). This layer shows neither increased porosity nor fractures and cracks, thus the cement paste shows very good adhesion to the porous EPS (Figure 7c–f).

SEM research by Ranjbar and Mousavi [45] showed low interphase bonds between EPS and cement paste, which was related to the hydrophobic and smooth surface of polystyrene granules [20]. Babu et al. [4] reported that the cement matrix surrounding Styrofoam particles may, with time, undergo microcracking and decomposition, allowing increased infiltration by water containing aggressive ions. It was also stated that EPS cements of greater particle sizes are characterized by higher water permeability. In the presented study, the applied EPS aggregates, obtained from mechanically processed polystyrene plates, have different surfaces to those of smooth polystyrene granules, so bonds between cement paste and EPS are particularly strong. 

**Figure 8:**
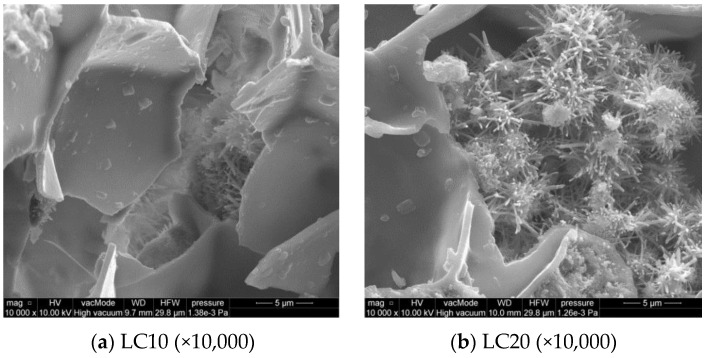
SEM microstructure of LC10 and LC20 concrete—EPS walls, fibrous structure of C–S–H phase and ettringite crystals at 10,000 times magnification. (**a**) LC10 (*×*10,000); (**b**) LC20 (*×*10,000).

The calcium silicate hydrate C–S–H phase, of different forms (Figure 8), is a very important component of cement paste. In most cases, the well-formed fibrous structure of C–S–H and sparse plates create a honeycomb structure, which crystallizes in empty spaces of the EPS. Figure 8b also shows an area rich in ettringite crystals. The morphology of the C–S–H phase indicates the Type I diamond classification, which reflects a well crystallized fibrous structure (Figure 8) [16].

**Figure 9:**
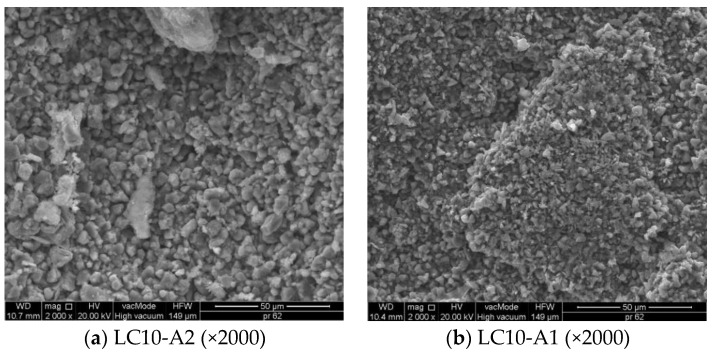
SEM microstructure of the coatings: (**a**) A2; (**b**) A1 at 2000 times magnification. (**a**) LC10-A2 (×2000); (**b**) LC10-A1 (×2000).

Figure 9 shows the SEM images of the coatings A1 and A2 on the LC10 surface.

The very good distribution of hydrophobic coatings inside the concrete structure was observed in SEM pictures (see Figure 9). Particles of polysiloxane gel, created during hydrolytic polycondensation, have variable sizes. The size of particle generated from tetramethoxysilane (Figure 9a) is greater than A1 and varies between 2 μm and 14 μm. However, particles of gel generated from silane (A1) have dimensions in the range 0.4–3 μm (Figure 9b). 

Similar differences in particle size of polysiloxane gel in the structure of mortar containing light aggregate waste were also observed [32]. Particles of methyl silicon resin that were too large were unable to penetrate the porous structure of the mortar, but settled on the sample surface, creating the thick coating, which cracked during dehydration and reduced the frost resistance of material [32]. The arrangement and size of particles, as presented in Figure 9, secured the analyzed LC with EPS against chemical corrosion and frost. Discontinuities, cracks, or tight concentration of hydrophobic coating were not observed.

## 4. Conclusions

The main purpose of this study was to determine the efficiency of hydrophobization of EPS concrete with the application of coating agents based on silane and tetramethoxysilane. Both applied hydrophobic agents are commercially available; however, due to their different chemical base and properties, they present different influences on securing the coated surfaces and structures of LC composites. Our studies aimed to indicate which of the two tested agents would be more efficient in the protection of LCs against severe environmental conditions, compared to standard concrete. 

Based on the analysis of the obtained results, the following conclusions are proposed:

1. The EPS addition to concrete influences the properties of the tested concrete samples. Application of EPS reduces strength characteristics, density, and frost resistance. The frost resistance of LC10 and LC20 after 50 F–T cycles decreases by 18 and 25 times compared to reference concrete. The EPS also increases porosity, absorbability, and water vapor permeability.
2. Hydrophobization allows the effective protection of all tested types of concrete, using both silane and tetramethoxysilane.
3. Application of EPS and organosilicon affects the wettability and adhesive properties of concrete, defined by SFE and CA. The lowest value of SFE, thus the weakest adhesive characteristics, is determined for LC hydrophobized by tetramethoxysilane.
4. A significant decrease in wettability and adhesive properties (SFE) is observed after surface hydrophobization of concrete, which indicates the high efficiency of the hydrophobization process.
5. Hydrophobization by silane agent results in significantly higher mass loss than after the application of tetramethoxysilane, after tests of reference concrete resistance against salt crystallization. These specimens contain porous additions in which pore crystallization of salt occurred. No visible damages of EPS concrete were observed.
6. Very good distribution of hydrophobic coatings inside the concrete structure was observed in SEM pictures. The size of particle generated from tetramethoxysilanes is greater than that from silane, and varies between 2 μm and 14 μm. However, particles of gel generated from silane (A1) have dimensions in the range 0.4–3 μm. Such arrangements and sizes of hydrophobic coating sufficiently protect LCs with EPS from frost and chemical corrosion.
7. The highest efficiency of EPS concrete hydrophobization was obtained after tetramethoxysilane application, while the performance of silane coating was weaker.

## Figures and Tables

**Table 1:** The basic characteristics of hydrophobizing agents.

Property	Agent A1 (Silane)	Agent A2 (Tetramethoxysilane)
Density (20 °C), kg/dm^3^	~0.94	~0.96
Appearance	White liquid	Clear, colorless, or slightly yellow liquid
Viscosity (20 °C), mPa·s	~15	~17
Active components, %	50	55
pH value (20 °C)	6–8	>13

**Table 2:** Chemical composition of cement clinker, %.

Compound	CaO	SiO_2_	Al_2_O_3_	Fe_2_O_3_	MgO	Na_2_O	K_2_O	Na_2_O_e_
Content	63.65	20.89	4.29	3.55	1.33	0.23	0.47	0.61

**Table 3:** Physical and mechanical properties of cement.

Le Chatelier, mm	Specific Surface, cm^2^/g	Specific Gravity, kg/dm^3^	Initial Setting Time, min	Heat of Hydration, J/g **	2-Day Compressive Strength, MPa	28-Day Compressive Strength, MPa
0.7	3821	3.07	158	305	28.9	55.6

** Heat of hydration measured after 41 h with the use of semi-adiabatic method.

**Table 4:** Sieve analysis of the fine and coarse aggregates, %.

Sieve Size, mm	Sand 0–2 mm	EPS 0–2 mm	Gravel Aggregate 2–16 mm
16	100	100	100
8	100	100	53.2
4	100	100	28.2
2	95.2	100	6.8
1	79.7	–	–
0.5	41.1	–	–
0.25	9.1	–	–
0.125	1.0	–	–

**Table 5:** Composition of concretes without/with recycled polystyrene.

Concrete Constituents	Unit	LC0	LC10	LC20
Portland cement CEM I 42.5 R	kg/m^3^	374		
Sand 0–2 mm	kg/m^3^	772	696	618
EPS 0–2 mm	kg/m^3^	0	0.52	1.05
Total waste replacement ratio (by volume)	%	0	10	20
Gravel 2–16 mm	kg/m^3^	1154		
Superplasticizer	kg/m^3^	3.6		
Water	L/m^3^	148		

**Table 6:** Basic properties of the tested LC.

Type of LC/Descriptive Statistics		Volumetric Density	Total Porosity	Compressive Strength *f*_c,cube#150_	Flexural Tensile Strength *f*_ct,flex_
		kg/m^3^	%	MPa	MPa
LC0	Mean	2250	6.48	70.1	8.53
	SD	3.67	0.13	1.26	0.37
	CV (%)	0.16	1.96	1.79	4.32
LC10	Mean	1960	14.48	62.9	7.38
	SD	2.92	0.76	2.01	0.11
	CV (%)	0.14	5.25	3.20	1.47
LC20	Mean	2080	25.22	57.2	5.77
	SD	2.74	1.57	1.30	0.09
	CV (%)	0.13	6.21	2.28	1.64

SD—standard deviation, CV—coefficient of variation.

**Table 7:** Contact angles and standard deviation of standard and hydrophobized LC.

Type of LC	Water Contact Angle (°)	
	before F–T Tests	after F–T Tests
LC0	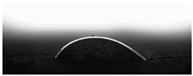	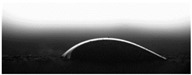
	CA = 44° SD = 0.72	CA = 40° SD = 0.82
LC0-A1	CA = 129° SD = 1.17	CA = 103° SD = 0.69
LC0-A2	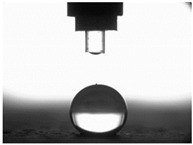	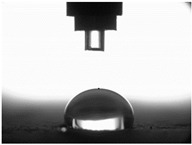
	CA = 136° SD = 0.61	CA = 110° SD = 1.58
LC10	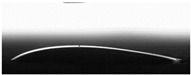	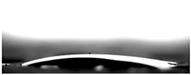
	CA = 34° SD = 0.79	CA = 11° SD = 0.78
LC10-A1	CA = 118° SD = 1.58	CA = 80° SD = 0.76
LC10-A2	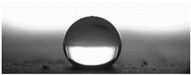	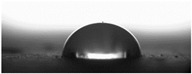
	CA = 130° SD = 0.82	CA = 89° SD = 0.99
LC20	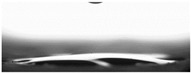	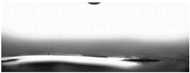
	CA = approx. 3° SD = 0.33	CA = approx. 2° SD = 0.12
LC20-A1	CA = 95° SD = 0.84	CA = 63° SD = 1.37
LC20-A2	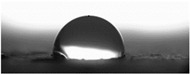	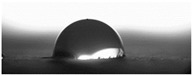
	CA = 102° SD = 0.65	CA = 75° SD = 1.09

## Data Availability

The data presented in this study are available on request from the corresponding author.

## Abbreviations

SFE	Surface free energy
CA	Contact angle
F–T	Freezing and thawing
LC	Lightweight concrete
EPS	Expanded polystyrene
SEM	Scanning electron microscope technique
LC0	Reference concrete without recycled polystyrene
LC10 or LC20	Concrete with 10% or 20% addition by volume of EPS
A1	Agent 1—silane
A2	Agent 2—tetramethoxysilane
w/c	Water/cement ratio
SD	Standard deviation
CV	Coefficient of variation
LC0-A1	Reference concrete coated with silane A1
LC0-A2	Reference concrete coated with tetramethoxysilane A2
AAS	Alkyl-alkoxy-silane
ITZ	Interphase transition zone
