# Correction: Barnat-Hunek, D., et al. Durability of Hydrophobic/Icephobic Coatings in Protection Lightweight Concrete with Waste Aggregate. *Materials* 2021, *14*, 101

**DOI:** 10.3390/ma14030620

**Published:** 2021-01-29

**Authors:** Danuta Barnat-Hunek, Jacek Góra, Marcin K. Widomski

**Affiliations:** 1 Faculty of Civil Engineering and Architecture, Lublin University of Technology, Nadbystrzycka 40, 20-618 Lublin, Poland; d.barnat-hunek@pollub.pl (D.B.-H.); j.gora@pollub.pl (J.G.); 2 Faculty of Environmental Engineering, Lublin University of Technology, Nadbystrzycka 40 B, 20-618 Lublin, Poland

The authors wish to revise in the main body text of 2.1 Materials, page 3 [1], due to the presence of incorrect information.

The correct text is shown below:

Both applied hydrophobizing agents were produced by Evonik Operations GmbH, Business Line Silanes, Rodenbacher Chaussee 4 63457, Hanau, Germany. Trade names of used preparations are as follows: A1—Protectosil^®^WS 405; A2—Protectosil^®^WS 808. The preparations were supplied by the distributor in Poland: CFI World S.A., Robakowo.

The previously given text is shown below:

Both applied hydrophobizing agents were produced by CFI World S.A. 62-023 Robakowo, Poland, based on ingredients from Evonik Industries, Business Line Silanes, Rodenbacher Chaussee 463457 Hanau-Wolfgang, Germany. Trade names of used preparations are as follows: A1—Protectosil^®^WS 405; A2—Protectosil^®^WS 808.

The authors would like to apologize for any inconvenience caused to the readers by these changes.
